# Improvements in Working Rubber

**Published:** 1874-12

**Authors:** 


### Correspondence.

### IMPROVEMENT IN WORKING RUBBER.

Mr. Editor:

An article with the above caption in the October number of the Register, has attracted my attention, and I fear that by it some one may be led to erroneous conclusions and possibly to actions that may result in getting them into trouble, not simply with the Vulcanite Company, but with the Courts as well.

The object, of the writer of the article alluded to, is undoubtedly a good one, viz., to make known a method whereby rubber plates may be made without infringing upon the Cummings' patent, and the rights of the Rubber Co., under that patent. But he is proceeding upon an entirely wrong assumption, and is evidently writing without a full knowledge or comprehension of the facts in relation to the subject; and his course leads him to a conclusion equally wrong.

With the object of explaining the true position of the matter at present, and of warning any who may have adopted the suggestion of the above writer, and with no disposition to attack the writer of the above article, I submit the following:

The article in question asserts that "it has long been conceded that the Cummings' claim is not for the application of rubber to dental purposes, but for the method of working it specified in the reissue No. 1904." The first part of this assertion is correct, viz., that the Cummings' patent does not cover the application of rubber to dental purposes. That would be scarcely a patentable improvement. "Dental purposes" is quite too indefinite a term, and the thing would be incapable of demonstration as required by the patent laws. It might mean a rubber plate, a rubber dental syringe, a rubber instrument handle, or spittoon funnel, or any one out of a dozen other things for which rubber may be used for "dental purposes." One, and one only, of all these is covered by Cummings' patent; and the simple perusal of the two lines of his claim will show what that is; viz., "the plate of hard rubber or vulcanite, or its equivalent, for holding artificial teeth, substantially as described." It becomes then simply a question of how the Courts will construe the claim, and what they will consider to be included in it. The first case in which the patent was brought into litigation was the Wetherbee case; and in that decision Judge Clifford held that the words "substantially as described" so enlarged the claim as to include the process by which the plate was constructed. But the Courts have since held that a process can not be included in the same patent as a product or result; and the evidence in the recently decided Smith case proved conclusively that the process was old and well known. The line of argument adopted bythe company in that case was radically different from that in the Wetherbee case; they have now abandoned all claim to the process, as a claim that they know can not be sustained, and rest their case upon the claim that the product, that is the plate of vulcanite for holding teeth, was a new and useful improvement, and was a patentable invention without reference to the process by which the result is reached. This is precisely the question now at issue in the Supreme Court. Dental plates being old, the process being old, and the material being old, is the product, the vulcanite dental plate a patentable invention is now the only question in regard to the Cummings' patent.

In conclusion I will remark that the process referred to is not original even as applied to rubber with the author of the article in the October REGISTER. If he will turn to the Dental Cosmos for April, 1874, he will there find an extract from the British Journal of Dental Science, in which the same is described in words so similar to his own, that it would seem that the writer must have had them before him when he wrote, nor is he correct in ascribing the method as applied to vulcanizable gutta percha to Dr. W. H. Eames. To Dr. E. Hale, Jr., the credit of that suggestion is due; but the process originated with one Slayton, who as long ago as 1856 constructed plates of gutta percha (unvulcanized), manipulating it exactly as now described, with the exception of the vulcanizing process. That a rubber plate can be constructed by this process is no doubt true; that it may possess some advantages over the ordinary method is possible; but that it will avoid the Cummings' patent or the claims of the Rubber Co., no one who has been a close student of the arguments, evidence, and decisions in re- to the Cummings' patent will mantain.

Such being the case, it will readily be seen that so long as the patent is sustained, a rubber plate however made will infringe it; and any one thinking to avoid it by varying the process, will find himself mistaken. Neither should any person under injunction think to escape by setting up this defence in case proceedings are commenced against him for violating the injunction.

